# A Simple Model to Study Mosaic Gene Expression in 3D Endothelial Spheroids

**DOI:** 10.3390/jcdd11100305

**Published:** 2024-10-02

**Authors:** Lucinda S. McRobb, Vivienne S. Lee, Fahimeh Faqihi, Marcus A. Stoodley

**Affiliations:** Macquarie Medical School, Faculty of Medicine, Health, and Human Sciences, Macquarie University, Sydney, NSW 2109, Australiamarcus.stoodley@mq.edu.au (M.A.S.)

**Keywords:** endothelial cells, adeno-associated virus, spheroid, vascular malformations

## Abstract

Aims: The goal of this study was to establish a simple model of 3D endothelial spheroids with mosaic gene expression using adeno-associated virus (AAV) transduction, with a future aim being to study the activity of post-zygotic mutations common to vascular malformations. Methods: In this study, 96-well U-bottom plates coated with a commercial repellent were seeded with two immortalized human endothelial cell lines and aggregation monitored using standard microscopy or live-cell analysis. The eGFP expression was used to monitor the AAV transduction. Results: HUVEC-TERT2 could not form spheroids spontaneously. The inclusion of collagen I in the growth medium could stimulate cell aggregation; however, these spheroids were not stable. In contrast, the hCMEC/D3 cells aggregated spontaneously and formed reproducible, robust 3D spheroids within 3 days, growing steadily for at least 4 weeks without the need for media refreshment. The hCMEC/D3 spheroids spontaneously developed a basement membrane, including collagen I, and expressed endothelial-specific CD31 at the spheroid surface. Serotypes AAV1 and AAV2^QUADYF^ transduced these spheroids without toxicity and established sustained, mosaic eGFP expression. Conclusions: In the future, this simple approach to endothelial spheroid formation combined with live-cell imaging could be used to rapidly assess the 3D phenotypes and drug and radiation sensitivities arising from mosaic mutations common to brain vascular malformations.

## 1. Introduction

The use of 3D spheroids to study tumor cell biology has expanded exponentially in the last 30 years, yielding valuable information on cellular differentiation, cell–cell and cell–matrix interactions, and therapeutic responses in this enhanced physiological niche [1]. Excluding studies of tumor vasculature, the use of 3D cell culture in endothelial cell biology has been far less actively explored but continues to expand our understanding of endothelial cell behavior in this type of microenvironment. Korff and Augustin first established 3D spheroids in 1998 from non-transformed endothelial cells (ECs) [2]. They demonstrated in a 3D context that ECs developed specific morphological and phenotypic characteristics associated with endothelial maturity, typically lost when cultured under standard 2D monolayer conditions. Moreover, 3D culture and the enhanced cell–cell contact, rather than cell-to-dish contact, increased the expression of survival factors and prolonged cell maintenance during extended culture in the absence of growth factors. In these spheroids, the ECs at the center underwent early apoptosis, eventually leaving an acellular core. The cells at the surface differentiated into a predominantly quiescent phenotype expressing mature endothelial markers, more reflective of the endothelium in vivo [3]. Korff and Augustin proceeded to use their endothelial spheroids for 3D angiogenic sprouting assays [4], eventually improving the consistency of this assay with the use of immortalized HUVEC [5]. The team subsequently produced mixed spheroids incorporating vascular smooth muscle cells (VSMCs) to demonstrate the additive effects of co-culture on the endothelial quiescence and muted growth factor reactivity [6]. Mixed spheroids have since been used for various purposes. For example, mixed spheroids of ECs, pericytes, and astrocytes have been used to study blood–brain barrier formation and drug transcytosis [7]. Recently, Nagaishi et al. produced mixed spheroids of ECs, VSMCs, and fibroblasts, and they used these in 3D printing to create vascular tubules for the study of vascular calcification [8]. There is no doubt that mixed spheroids provide enhanced physiological relevance; however, their complexity limits high-throughput analysis. Simple endothelial spheroids therefore provide an easily reproducible first-pass model and an independent set of characteristics that bridge the knowledge gap between 2D and in vivo environments.

We are interested in investigating the mutations that drive cerebrovascular disorders such as brain arteriovenous malformations (bAVMs). In sporadic bAVMs, mutations arise post-zygotically in the endothelium, leading to mosaic gene expression. A variety of driver mutations have been identified, with the most common occurring in oncogenic *KRAS* (Kirsten rat sarcoma viral oncogene homologue) [9,10,11]. To investigate the mosaic expression of bAVM driver mutations, we therefore aimed to first establish a simple reproducible 3D spheroid model that could be easily adapted to assess the multiple mutation types relevant to bAVMs, or other vascular disorders, in the future.

In this study, we examine two commonly used immortalized endothelial cell lines, one from umbilical veins and one from brain vessels, for their ability to form robust and reproducibly sized spheroids using a simple approach involving spontaneous aggregation in cell-repellent, U-bottom, 96-well plates. We further examine the ability of endothelial spheroids to take up adeno-associated viral (AAV) serotypes and establish stable enhanced green fluorescent protein (eGFP) expression with a mosaic distribution. We investigate the utility of a live-cell imaging platform to enhance our throughput. Lastly, we investigate the utility of various commercial agents for monitoring cell viability and death in these spheroids in future studies.

## 2. Materials and Methods

### 2.1. Cell Culture

Immortalized human cerebral microvascular endothelial cells, hCMEC/D3 (CELLutions Biosystems Inc., Toronto, ON, Canada), were cultured as previously described [12,13] in EBM-2 medium (Lonza, Basel, Switzerland) supplemented with 5% fetal bovine serum, 1% penicillin/streptomycin, 10 mM HEPES (Life Technologies, Grand Island, NY, USA), and 1 ng/mL human basic fibroblast growth factor (Sigma-Aldrich, North Ryde, Australia). Immortalized HUVEC-TERT2 (CRL-4053TM, ATCC) were cultured in EBM-2 medium (Lonza) supplemented with an EBM-2 endothelial growth kit (Lonza, #CC4176). The cells grew optimally in flasks coated with 100 µg/mL type I collagen (rat tail, Sigma-Aldrich, #354236). All the cells were grown at 37 °C in 5% carbon dioxide and were passaged at 90% confluence with 0.1% trypsin-EDTA (Life Technologies).

### 2.2. Spheroid Formation

U-bottom, 96-well plates with a non-adherent surface (Nunclon Sphera 96-well, Nunclon Sphera-Treated, U-Shaped-Bottom Microplate #179425; Thermo Fisher Scientific, Waltham, MA, USA) were used for the spheroid formation. The trypsinized cells were counted before seeding at defined cell densities. Standard complete growth media, as described above, was used for each cell line. Rat tail type I collagen dissolved in phosphate-buffered saline (PBS) was added to the full growth medium for the formation of HUVEC-TERT2 spheroids at specified concentrations (0–10 µg/mL). Stable spheroid formation was confirmed when the spheroids could be gently pipetted and moved between wells without altering the spheroid morphology. Once seeded, all the spheroid experiments were performed without the addition of fresh medium (or medium exchange).

### 2.3. AAV Construction

All the recombinant AAV constructs and virus suspensions were created and supplied by VectorBuilder (Chicago, IL, USA). A mammalian gene expression vector was used, encoding eGFP driven by a CMV promoter (vector name: pAAV[Exp]-CMV>EGFP:WPRE; Vector Builder ID VB010000-9394npt). This was packaged into 3 viral constructs with varying serotypes: AAV1, AAV5, and AAV2^QUADYF^. Titers of ~4–5 × 10^11^ GC/mL were supplied. Viral particles were used to transduce the cells in 2D or 3D at a multiplicity of infection (MOI) between 1000 and 2000, respectively. Viral particles were diluted in PBS before the cell or spheroid addition to allow dispersed cellular uptake in the wells. Viral particles were added to full growth medium and could be left for extended periods without observations of cell death or toxicity.

### 2.4. Viability and Cell Death Assays

Hydrogen peroxide (H_2_O_2_) was used to stimulate apoptosis and was diluted in a suitable volume of PBS (10 µL) before addition to the spheroids in full growth medium to achieve final concentrations of 0, 0.1, 1 and 10 mM. H_2_O_2_ was added on day 3 after seeding (at a density of 500 cells/well) when the spheroids were stably formed. Dyes were added 24 h after H_2_O_2_ exposure. The spheroids were irradiated with a final dose of 15 or 25 Gy by a 6 MV linear accelerator (Elekta Synergy, Crawley, UK) at Macquarie University Hospital (Sydney, Australia), as previously described [14,15]. The spheroids were irradiated 3 days after spheroid seeding. The sham controls were treated identically but were not irradiated. All the spheroids (control and irradiated) were transferred into a single new 96-well U-bottom plate once the irradiation was completed, as the spheroids could not be assembled in the same plate prior to irradiation. Dye addition and imaging were performed 1–3 days after radiation exposure as specified.

ANV-FITC (1/100 dilution, Invitrogen, Waltham, MA, USA; A13199), propidium iodide (PI) (final concentration of 0.25 µg/mL, BD Pharmingen, Franklin Lakes, NJ, USA; #556463) and 7-aminoactinomycin D (7-AAD) (final concentration of 0.25 µg/mL, Eugene, OR, USA, A1310) were added 2 h prior to imaging. Calcein AM Blue Viability Dye (Invitrogen, #65-0855-9) was dissolved in dimethylsulfoxide (DMSO), added at a final concentration of 10 µg/mL, and imaged 30 min after addition. The CyQUANT Direct Cell Proliferation Assay was used as described by the manufacturer (Life Technologies, Grand Island, NY, USA; C35011). The autofluorescence was monitored in the control, unstained spheroids subject to the same treatments.

### 2.5. Microscopy and Image Analysis

Static imaging was routinely performed with an EVOS FL Digital Inverted Microscope equipped with Phase, Blue (DAPI), Red (Texas RED), and Green (FITC) filter sets (Invitrogen, AMF4300, AMEFC4300). The area analysis of the phase-contrast images was performed manually using NIH Image J v1.54f [16]. Briefly, EVOS FL images at 100× magnification were imported into Image J and masks were created by a modification of Lacalle et al. [17]. Essentially, an ROI was created around each spheroid (8-bit images) and a preliminary mask created using the “Find edges” process. The processed image was then made binary, dilated and had any holes filled to ensure a single mask around each spheroid. If necessary, the images were then processed by eroding to ensure the defined mask was representative of the spheroid area. The area was then determined using “Analyze particles” set to filter out small cellular debris, with the scale set to convert pixels to µm^2^ for each magnification.

The fluorescence in the EVOS FL images was determined using Image J [16]. The images were color deconvoluted into their appropriate RGB file. A rectangular ROI was then created around the spheroid of a standard size (replicated for all the images) and the mean gray value recorded. A replica ROI was prepared on the same image to record the background fluorescence and this value was subtracted from the original. An Incucyte SX5 Live-Cell Analysis System (Sartorius, Göttingen, Germany) was used to digitally monitor and measure the spheroid area by “mask” creation and green (FITC, eGFP)/far red (PI) fluorescence in real-time. The raw data were analyzed with instrument-associated software then exported for analysis.

### 2.6. Immunostaining and Confocal Microscopy

Immunocytochemistry was performed on the hCMEC/D3 spheroids. All the staining steps were performed in the U-bottom, non-adherent plates where they were formed. First, the culture media was exchanged three times with PBS to wash the spheroids, prior to 30 min of fixation with 4% paraformaldehyde. The spheroids were then washed by PBS exchange before permeabilization with 0.1% Triton X-100 for 15 min. The spheroids were washed a further three times prior to blocking with 1% bovine serum albumin (BSA) and donkey serum (5%) in PBS for 1 h. Antibodies against collagen I (rabbit polyclonal, AF0134, Affinity Biosciences, Cincinnati, OH, USA) and CD31/PECAM-1 (mouse monoclonal, sc-376764, Santa Cruz, Dallas, TX, USA) were used with species-specific AlexaFluor647-conjugated secondary antibodies (Life Technologies, Grand Island, NY, USA) to visualize the basement membrane and endothelial surface, respectively, by staining overnight at 4 °C. The control sections incubated with rabbit IgG (Santa Cruz Biotechnologies, Dallas, TX, USA) or mouse IgG (BD Biosciences) showed no reactivity. The nuclei were counterstained with 4′,6-diamidino-2-phenylindole dihydrochloride (DAPI, 5 μg/mL, Life Technologies). Two-dimensional and Z-stack images were taken under fixed parameters using a Zeiss LSM800 Confocal Microscope (Jena, Germany). The Z-stack images were taken with the First/Last operational mode; the range and number of Z-stacks was adjusted based on spheroid size. Images were acquired from three channels: ex358 nm/em461 nm for DAPI (nuclei); ex 495/em519 nm for eGFP; and ex650 nm/em665 nm for AF-647 (collagen I, CD31).

### 2.7. Statistical Analysis

Prism version 9.3.0 (GraphPad, La Jolla, CA, USA) was used to graph the data and generate statistical analyses. All the data from single intraplate replicates are shown as the mean ± standard deviation (SD), while data from independent experiments are shown as the mean ± standard error measurement (SEM). One-way ANOVA or two-way ANOVA was used with Tukey’s post hoc analysis as specified in the figure legends.

## 3. Results

### 3.1. Spheroid Formation in hCMEC/D3

Two endothelial cell lines were assessed for their ability to form spheroids using a simple aggregation method. Immortalized human cerebral microvascular endothelial cells (hCMEC/D3) and human umbilical vein endothelial cells (HUVEC-TERT2) were seeded in defined cell numbers in 96-well, U-bottom plates coated with a repellent surface. The cell aggregation was monitored daily with an EVOS FL Digital Inverted Microscope (Invitrogen) or Incucyte SX5 Live-Cell Analysis System (Sartorius).

**Figure 1:**
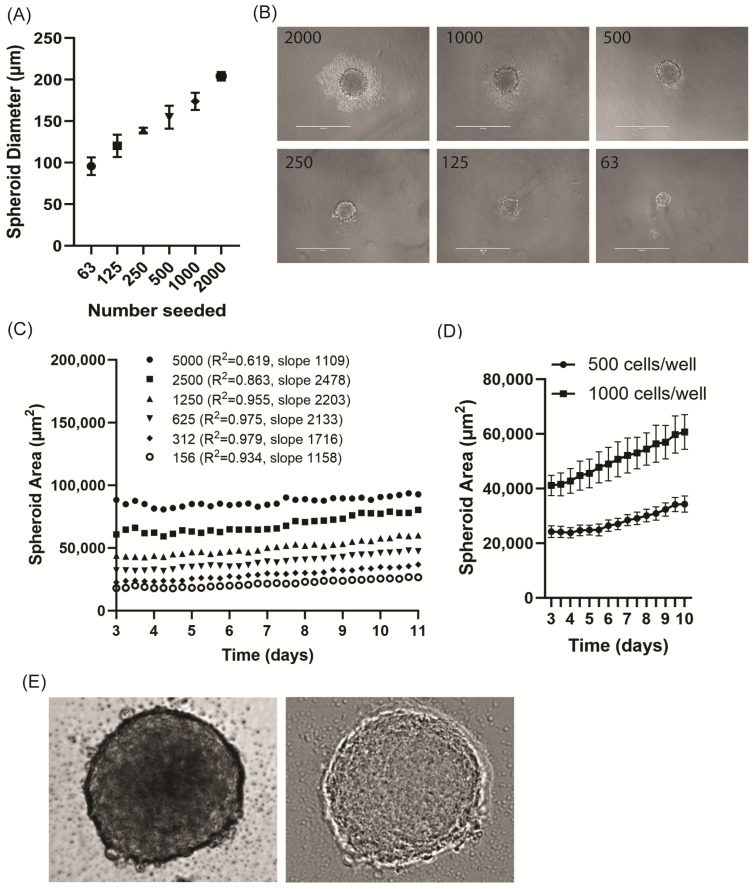
Characteristics of spheroid formation in hCMEC/D3 cells. (**A**) hCMEC/D3 were seeded at densities between 63 and 2000 cells per well, images were taken on an EVOS FL Inverted microscope and diameters measured using Image J (day 7, N = 4 technical replicates, SD shown). (**B**) Representative phase-contrast images of endothelial spheroids acquired on an EVOS FL Inverted Microscope (day 7); scale bar = 400 µm. (**C**) hCMEC/D3 were seeded at densities between 156 and 5000 cells per well and immediately placed in an Incucyte SX5 Imaging System for live-cell imaging analysis. The mean area (µm^2^) was determined for 6 technical replicates per cell seeding density (SD not shown for clarity). Simple linear regression was performed on the data from day 3 to 11 (after stable spheroids formed) to determine the best fit (slope = growth rate at µm^2^/day) and goodness of fit (R^2^) for each seeding density. (**D**) Cells seeded at 500 cells/well or 1000 cells/well demonstrated stable, reproducible spheroid formation once the cells had pooled to the bottom of the U-bottom plates and aggregated between days 2 and 3 (mean ± SEM of N = 4 independent experiments). (**E**) Representative brightfield (**left**) and phase-contrast (**right**) images acquired using the live-cell imaging platform show a diffuse pattern inside the spheroid with a clear delineation of the dense spheroid border.

The hCMEC/D3 cells aggregated spontaneously and formed stable spheroids within 3 days (Supplementary Video S1). The spheroids formed freely without adherence, sitting independently at the bottom of the U-shaped well. The spheroids were robust. Once formed, the hCMEC/D3 spheroids could be readily pipetted and transferred between wells without degradation or disruption. The cells seeded at densities between 62 and 2000 cells per well formed spheroids with mean diameters ranging from 100 to 200 µm after day 7, demonstrating a linear relationship to the seeding density (Figure 1A,B).

In the Incucyte SX5, the spheroid growth could be monitored over time using live-cell imaging. Mask formation was used to determine the spheroid area (Supplementary Figure S1). The mean spheroid area increased slowly from day 3 (once spheroids had formed), with the linear growth rates dependent on the seeding density up to 2500 cells per well (Figure 1C). Further independent experiments were performed with cells seeded at densities of 500 and 1000 cells per well, where the good reproducibility of spheroid formation was observed within experiments (Supplementary Figure S2) and between experiments (Figure 1D). In the Incucyte SX5, the spheroids demonstrated formation of a clearly delineated, optically dense border (Figure 1E).

### 3.2. Spheroid Formation in HUVEC-TERT2

**Figure 2:**
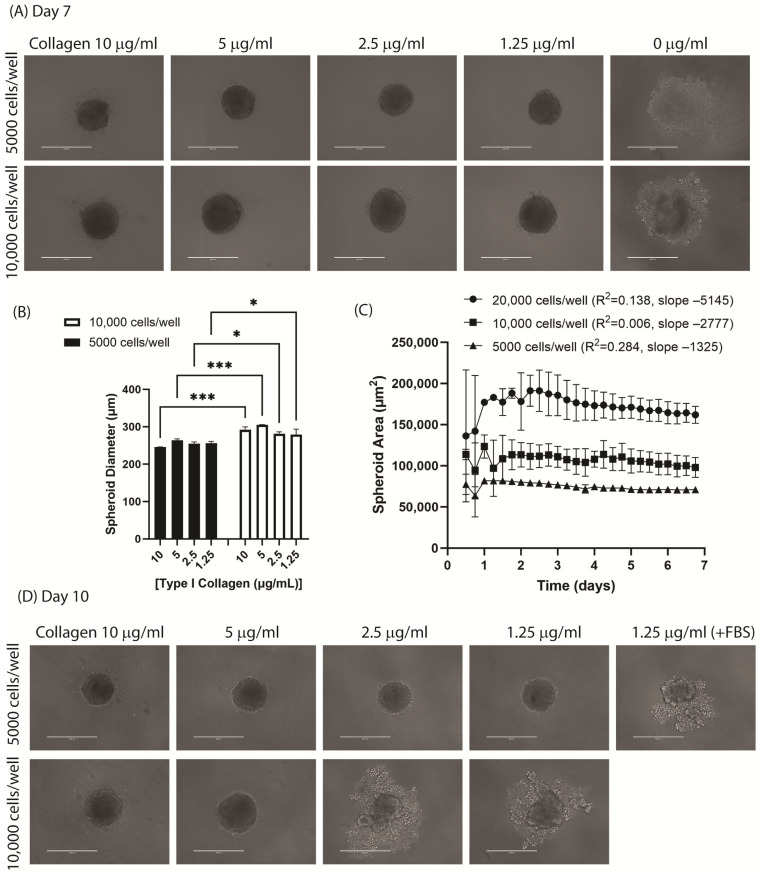
Characteristics of spheroid formation in HUVEC-TERT2 cells. (**A**) HUVEC-TERT2 were seeded at densities between 5000 and 20,000 cells per well in the presence of rat tail type I collagen (1.25–10 µg/mL). Representative phase-contrast images of endothelial spheroids were acquired on an EVOS FL Inverted Microscope (day 7). Absence of collagen resulted in failure to form an aggregated sphere (**right**). (**B**) Spheroid diameters were measured using Image J (day 7) and statistical comparisons performed between the number of cells seeded and collagen concentrations using two-way ANOVA (Tukey’s post hoc test: * *p* < 0.05, *** *p* < 0.01) (N = 3 independent experiments, mean ± SEM. (**C**) HUVEC-TERT2 were seeded and placed in a live-cell analysis system and the mean area (µm^2^) monitored over 7 days (N = 4–6 technical replicates per cell seeding density, mean ± SD). Simple linear regression was performed on data from day 3 to 7 to determine the best fit (slope = growth rate at µm^2^/day) and goodness of fit (R^2^) for each seeding density. (**D**) Representative phase-contrast images of spheroids taken on day 10 post-seeding demonstrating spontaneous spheroid rupture in cells seeded at 10,000 cells per well, and in cells seeded at 5000 cells per well after addition of fetal bovine serum (FBS) to induce rupture. Scale bars, 200 µm.

The HUVEC-TERT2 cells were grown in fully supplemented growth medium and seeded at densities between 250 and 20,000 cells per well. The cells settled at the center of each U-bottom well, but even with a very high cell density, they did not spontaneously form solid spheroids within 2 weeks after plating (Figure 2A, last column; Supplementary Video S2). Spheroids could only be formed in the presence of soluble rat tail type I collagen when included in the growth medium at the time of seeding (Figure 2A; Supplementary Video S3). Other methods commonly employed to stimulate spheroid development, including use of alternative growth media, increasing the serum concentration, plate centrifugation, and addition of 1–2 mg/mL methylcellulose (a thickening agent known to assist in cell aggregation) [18], also failed to stimulate spheroid formation in this cell line.

The spheroid diameters were dependent on the HUVEC-TERT2 seeding density but not significantly on the collagen concentration in the medium (Figure 2B). The mean spheroid areas were 256 ± 7.5 and 288 ± 13.1 when seeded at 5000 or 10,000 cells per well, respectively (5000 vs. 10,000, *p* < 0.0001). Using live-cell imaging, the spheroid formation with HUVEC-TERT2 appeared to stabilize by day 3, after which the spheroid area registered a steady but slow decline in the absence of media exchange, confirmed by simple linear regression analysis of the slopes between day 3 and day 7 (Figure 2C). Subsequent monitoring of the spheroid behavior with extended culture using the EVOS FL microscope demonstrated that the spheroids eventually ruptured, with cell escape evident on the outside of each spheroid between days 10 and 11. The time of spontaneous rupture post spheroid formation was accelerated by a higher seeding density or lower collagen content. Rupture could also be stimulated by medium exchange or addition of serum (Figure 2D), suggesting that rupture was most likely due to ongoing cell proliferation rather than cell death causing spheroid disintegration.

### 3.3. Transduction Efficiency of AAV Serotypes in Endothelial Cell Lines and Spheroids

**Figure 3:**
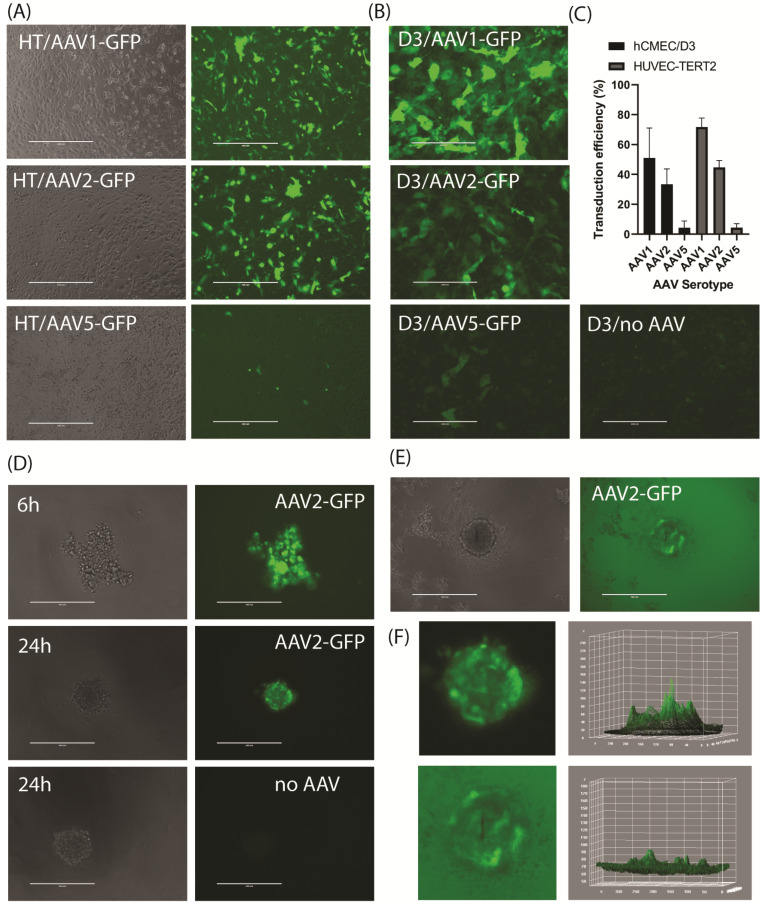
Transduction of endothelial cells with AAV serotypes produces mosaic eGFP expression in spheroids. Representative images of HUVEC-TERT2 (**A**) and hCMEC/D3 (**B**) in 2D culture transduced with AAV serotypes (AAV1, AAV2^QUADYF^, AAV5; MOI 1000–2000) were left for 4–5 days without media exchange to allow eGFP expression (green). (**C**) Transfection efficiency was determined in 2D cultures for all three serotypes in both HUVEC-TERT2 and hCMEC/D3 cells in 3 independent experiments (mean ± SEM). (**D**) hCMEC/D3 cells were transduced with AAV2^QUADYF^ in 2D culture and after 3 days were trypsinized and re-seeded at 500 cells/well into U-bottom 96-well plates for spheroid formation. (**E**) Spheroids were transduced 3 days after seeding into U-bottom plates and fluorescence monitored for 3 days. (**F**) Enlarged images of eGFP-expressing spheroids from (**D**,**E**) with associated 3D surface plots created in Image J. Transduction with AAV before spheroid formation led to a consistent pattern of expression throughout, while transduction after spheroid formation produced mosaic patterns with clustered patches of eGFP expression that appeared to be predominantly on the surface.

Three AAV serotypes, AAV1, AAV2^QUADYF^, and AAV5, were tested for their ability to transduce the two cell lines using an eGFP-tagged control construct. The AAV1 and AAV5 serotypes have previously been described to efficiently transduce endothelial cells [19,20], while the AAV2^QUADYF^ variant was designed with a capsid mutation to provide efficient uptake and specific expression in ECs [21]. For AAV1 and AAV2^QUADYF^, 30–70% eGFP-positive cells could be obtained 3–4 days after transduction in 2D hCMEC/D3 and HUVEC-TERT2 cultures at a multiplicity of infection (MOI) of 1000–2000 (Figure 3A–C). The cells transduced with AAV5 demonstrated limited eGFP expression (<5%) in both cell lines (Figure 3A–C).

The AAV uptake and eGFP expression were then assessed in the 3D spheroids. First, the hCMEC/D3 cells were transduced in 2D cultures using the AAV1 and AAV2^QUADYF^ serotypes, and 3 days were allowed for eGFP expression. The cells were then trypsinized and seeded into U-bottom plates. The spheroid formation remained unaffected by the AAV infection and spheroids formed rapidly within 24 h (Figure 3D). Fluorescence could be observed throughout the spheroid (both centrally and peripherally) with eGFP expressed in a mosaic pattern, while the spheroids without AAV transduction demonstrated limited autofluorescence (Figure 3D,F).

Transduction of pre-formed hCMEC/D3 spheroids was then conducted. Viral constructs (1 × 10^9^ viral particles) were diluted in phosphate-buffered saline and added to each spheroid-containing well and incubated for 4 days. In these spheroids, the eGFP expression was also mosaic but more randomized, and transduction seemed to occur predominantly in peripheral cells in small clusters when analyzed by the EVOS microscope (Figure 3E,F). There was no evidence of spheroid disruption or spheroid growth in response to AAV addition. It should be noted that as we routinely did not perform media exchange or washing of the spheroids after the AAV addition, there was a significant contribution to background autofluorescence from the added viral mix using this methodology (Figure 3E,F).

### 3.4. Maintenance of eGFP Gene Expression and Expression of Endothelial Markers

**Figure 4:**
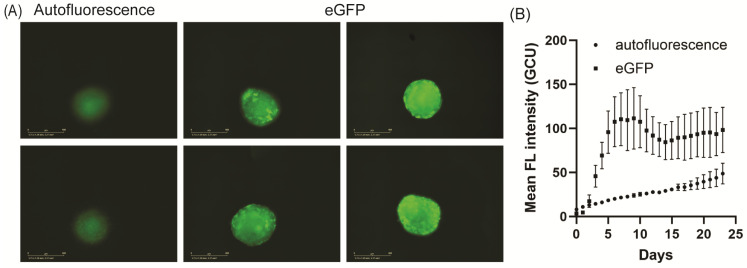
Transduction of endothelial spheroids with AAV serotypes produces mosaic eGFP expression. (**A**) Representative images of hCMEC/D3 spheroids with (**right**) or without (**left**) AAV ^QUADYF^ transduction after 23 days in culture. eGFP expression was random and mosaic in transduced spheroids; spheroids without eGFP showed low levels of autofluorescence at the center of the spheroid. Scale bar, 400 µm. (**B**) Intraplate assessment of spheroids transduced with AAV-eGFP or without AAV (N = 9 spheroids per group) showed that the total eGFP fluorescence between spheroids was high but heterogeneous, reflective of the random mosaic expression pattern observed (mean fluorescent intensity ± SD). eGFP expression increased rapidly from day 2, peaking at day 6, before remaining stable until at least day 23 (squares; end of the experiment), compared to the steady increase in autofluorescence observed in non-transduced spheroids (circles).

Further work was performed on the more robust hCMEC/D3 spheroids. We focused on transduction with AAV2^QUADYF^ as the optimal AAV for future experiments, and we used the Incucyte SX5 for continuous monitoring of the eGFP expression. hCMEC/D3 spheroids were cultured for extended periods in the Incucyte SX5 (up to 3–4 weeks) to monitor for the presence of extended mosaic gene expression. The eGFP expression was shown to increase dramatically from day 2 and peaked around day 6, with the mean intensity remaining high until at least week 3 (Figure 4A,B). The AAV-eGFP transduction was consistently high, but using the live-cell analysis system, we observed heterogeneity in the extent of the expression from each spheroid within the same experiment, consistent with the mosaic gene expression achieved and the random pattern of cellular distribution. The spheroids without AAV-GFP transduction showed low levels of autofluorescence at this excitation wavelength, which rose slowly with time, in line with the slow increase in the spheroid size over time. This background fluorescence could not be removed as the lowest exposure setting of 100 ms in the instrument was used. This autofluorescence tended to arise from the center of the spheroid, and it may represent the accumulation of lipofuscin, a molecule that accumulates in stressed cells with down-regulated lysosomal degradation, autophagy, or senescence [22,23].

**Figure 5:**
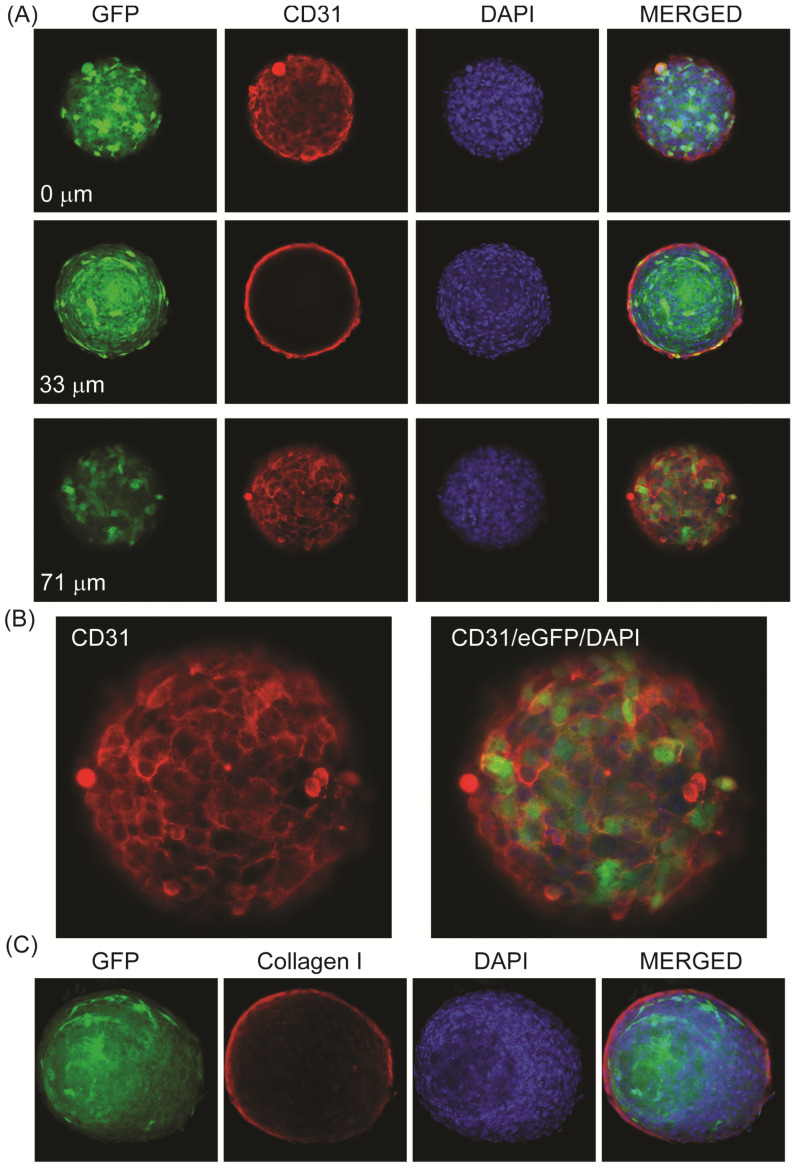
Confocal imaging of hCMEC/D3 endothelial spheroids. (**A**) Representative images of hCMEC/D3 spheroids collected using confocal microscopy and Z-stack imaging after 23 days in culture with AAV-eGFP transduction (AAV2^QUADYF^) and immunostaining for CD31. Top, middle and bottom images (separated by approximately 70 µm) through the stack are shown. eGFP (green) expression was random and mosaic in transduced spheroids and present both centrally and peripherally. The endothelial marker CD31 (AF647, red) was present at the spheroid surface and demonstrated the presence of flattened, cobblestone cells ((**B**), enlarged images). DAPI staining (blue) was used to stain the cell nuclei and to show the cell density in the spheroid core. (**C**) Representative images (middle of Z-stack) showing AAV-eGFP-transduced (green) spheroids immunostained with anti-collagen I antibody (AF647, red) (middle section of Z-stack shown). The area devoid of DAPI-positive cells indicates an acellular region in the core. Images were collected on a Zeiss LSM880 Confocal microscope, 20× objective.

Using immunostaining of the whole spheroids and the creation of Z-stacks with confocal microscopy, it was observed that eGFP expression was not only seen at the surface but also throughout the spheroid core in a mosaic pattern (Figure 5A–C; Supplementary Figure S3). Confocal imaging of eGFP was performed in the spheroids that had also been immunostained for endothelial specific markers. Figure 5A,B show the eGFP expression in the spheroids co-stained with CD31, a surface marker also known as platelet and endothelial cell adhesion molecule 1 (PECAM-1). The Z-stack images of the top, middle, and bottom of a representative spheroid show that CD31 expression was evident on the surface of the spheroids (Figure 5A,B; Supplementary Figure S4a) and also demonstrate that at the face of the spheroid, the cells were flattened and cobblestone, as found typically in 2D endothelial cultures and by Korff and Augustin in their original spheroid studies [2].

Other representative spheroids were immunostained with an anti-collagen I antibody (Figure 5C, Supplementary Figure S4b). Collagen I was also associated with the spheroid surface, consistent with its role in basement membrane formation. As noted earlier, hCMEC/D3 did not require collagen in the growth medium for aggregation and spheroid formation. This result shows that hCMEC/D3 could spontaneously produce collagen I, potentially contributing to the natural ability of this cell line to form 3D structures. Confocal imaging of the nuclear staining with DAPI also demonstrated that the cells within the spheroid remained densely packed after 3–4 weeks of culture (Figure 5A,C), although centralized acellular regions were not uncommon in these spheroids after extended culture (Figure 5C), likely due to the ongoing cell death in the core, as observed previously [2].

### 3.5. Monitoring Cell Viability in hCMEC/D3 Spheroids

Once formed, the hCMEC/D3 spheroids were robust, and with extended culture, were difficult to reproducibly trypsinize for cell counting. The cells on the exterior of 200 µm spheroids could be readily removed by 15–30 min incubation with 0.1% trypsin-EDTA at 37 °C; however, the core could be highly resistant to disruption after extended culture (not shown). Several dyes and reagents, as well as observations of spheroid morphology, were then used to assess the cell viability and cell death characteristics in these 3D spheroids. So that the validity of the dyes could be assessed as a potential future determinant of cell death or viability, the spheroids were also exposed to H_2_O_2_ to induce reactive oxygen species (ROS), cellular damage, and apoptosis using concentration ranges previously determined to stimulate apoptosis in 2D cultures [24].

For the viability assessment, we compared Calcein-AM, a dye used to assess cell viability as it can only be retained in the cytoplasm of live cells with active cytosolic esterases and without membrane disruption [25], with the CyQUANT Direct Cell Proliferation assay, which contains a proprietary dye for quantifying cell proliferation and cytotoxicity through measurement of the DNA content.

**Figure 6:**
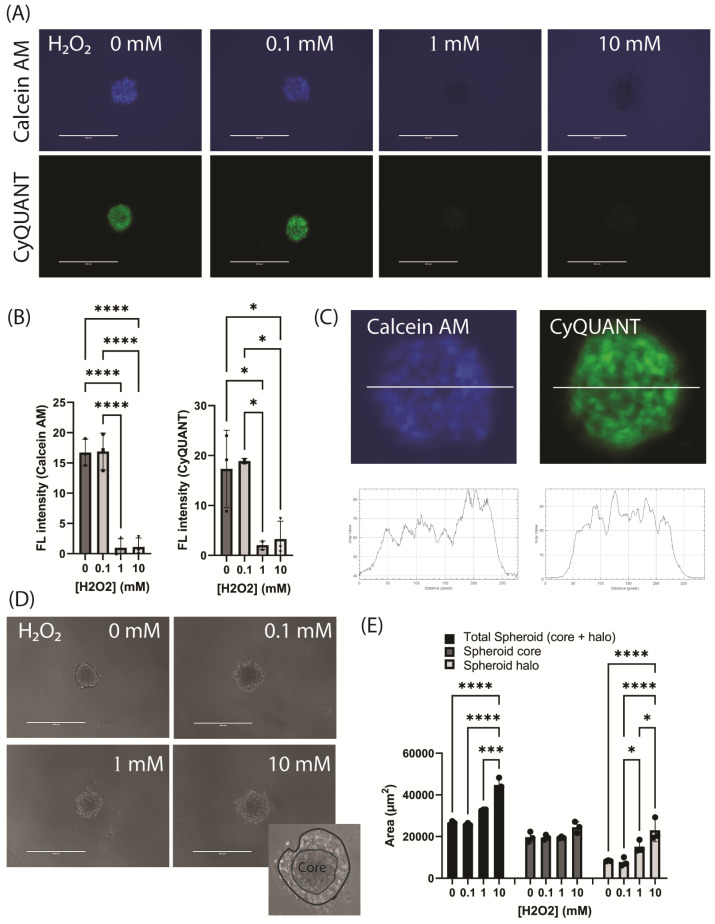
Cell viability and morphology changes in hCMEC/D3 treated with hydrogen peroxide. (**A**) Representative images of hCMEC/D3 spheroids stained with Calcein-AM (blue) or with the CyQUANT proliferation kit (green) after treatment with hydrogen peroxide (H_2_O_2_, 0–10 mM). (**B**) Fluorescence intensity was determined using Image J (N = 3 independent experiments, mean ± SEM). (**C**) Enlarged images of Calcein-AM- and CyQUANT-stained spheroids and profile plots (Image J) showing fluorescence distribution. (**D**) Representative brightfield images demonstrating the spheroid morphology in response to H_2_O_2_ treatment. Enlarged inset shows disintegration of the spheroid into the core structure with halo of cells. (**E**) Spheroid area (µm^2^) was determined using Image J and subdivided into regions encompassing the core and halo of loosened cells (N = 3 independent experiments, mean ± SEM). All the data were analyzed with one-way ANOVA (Tukey’s post hoc test; * *p* < 0.05, *** *p* < 0.001, **** *p* < 0.0001). Scale bars, 400 µm.

In the untreated spheroids formed by day 3, Calcein-AM was retained in the cells but was abruptly lost between H_2_O_2_ concentrations of 0.1 and 1 mM (Figure 6A–C). In the untreated spheroids formed by day 3, the CyQUANT dye uptake was similar, being present in the untreated spheroids and at low H_2_O_2_ concentration, but was abruptly lost at higher concentrations. The cellular pattern of staining, however, was sharper, consistent with its nuclear rather than cytoplasmic localization. (Figure 6A,C).

The spheroid viability was also assessed by monitoring the spheroid morphology using phase-contrast microscopy in the EVOS FL microscope (Figure 6D). Significant changes in the spheroid morphology were observed in response to H_2_O_2_ exposure. The spheroids initially appeared to become less compact, leading to an increase in the spheroid “area” unrelated to cellular proliferation or spheroid growth (Figure 6E). With higher concentrations of H_2_O_2_, the spheroids developed a “halo” of shed cells that led to a pseudo increase in “area”, as defined by the cell masking approaches used in Image J (Figure 6D,E). The core regions did not significantly decrease or increase in size despite this shedding of cells at the periphery, as the cells within the core became more loosely attached and the structure less compact (Figure 6D,E). The pattern of changes did, however, reflect the decrease in cell viability that was observed with the viability dyes. This morphology could also be observed and recorded in the Incucyte SX5; however, automated masking with the Incucyte software was problematic as the dead cell area could not be distinguished adequately from the remaining spheroid core.

### 3.6. Monitoring Cell Death in hCMEC/D3 Spheroids

Cell death in the 3D spheroids was assessed with or without H_2_O_2_ addition and was monitored using several death-detecting dyes. Early apoptotic events were examined using an Annexin V-FITC conjugate to target the exposure of the membrane phospholipid, phosphatidylserine, on the cell surface [26]. Propidium iodide (PI) and 7-aminoactinomycin D (7-AAD) were used as late apoptotic or necrotic markers. Both the PI and 7-AAD dyes fluoresce strongly once intercalated with DNA in the nucleus; however, both are impermeable to intact live cells [27,28].

**Figure 7:**
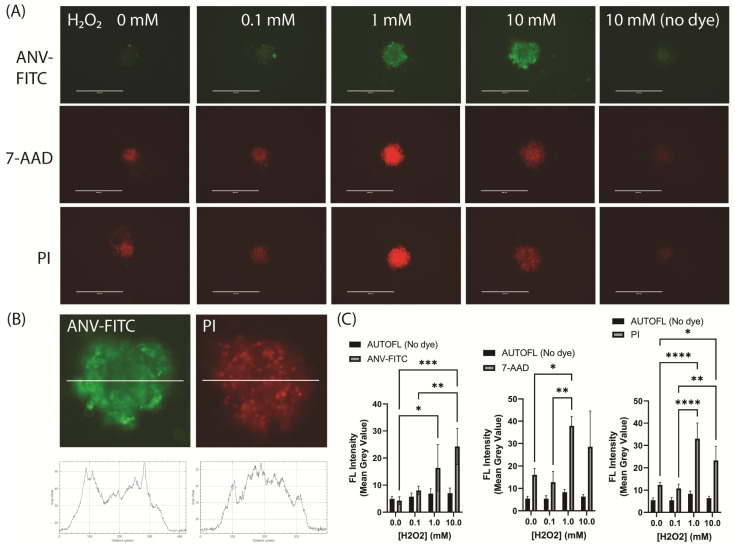
Effect of hydrogen peroxide on the cell death distribution in hCMEC/D3 spheroids. (**A**) Representative images of H_2_O_2_-treated spheroids stained with viability dyes. Three days after seeding the U-bottom plates, stable spheroids were treated with H_2_O_2_ (0–10 mM), and after 24 h, dyes were added to assess cell death. Images were acquired on an EVOS FL Inverted Microscope (scale bar = 400 µm). Spheroids were stained for 2 h with Annexin V-FITC (ANV-FITC, green), propidium iodide (PI, red), or 7-aminoactinomycin D (7-AAD, red). Autofluorescence in unstained cells was also observed and measured (**right** column). (**B**) Enlarged images of ANV-FITC and PI-stained spheroids demonstrating the relative distribution and pattern of fluorescence staining (central or peripheral) across each spheroid. Cross-sections of each spheroid were analyzed using Plot Profile in Image J. (**C**) Fluorescence was determined across 3 independent experiments using Image J for ANV-FITC, 7-AAD and PI-stained spheroids. Data were analyzed using two-way ANOVA (Tukey’s post hoc test; * *p* < 0.05, ** *p* < 0.01, *** *p* < 0.001, **** *p* < 0.0001). All the data are shown as the mean ± SEM.

ANV-FITC expression was detectable over and above the background autofluorescence. Limited FITC expression was evident in untreated spheroids at day 3–6 post-seeding; however, H_2_O_2_ increased the fluorescent intensity in a concentration-dependent manner (Figure 7A,B). ANV-FITC staining was predominantly localized to peripheral cells, with a pattern reflecting membrane rather than nuclear staining, consistent with phosphatidylserine at the cell surface being its molecular target (Figure 7B).

The PI and 7-AAD uptake contrasted significantly to that of ANV-FITC. There was evidence of PI and 7-AAD nuclear uptake in the untreated spheroids that was scattered sporadically throughout the spheroid, but it was found to be primarily centralized, consistent with the majority of spheroid studies that show cells within the core of 3D structures are typically apoptotic or necrotic (Figure 7A–C). In response to H_2_O_2_, both the PI and 7-AAD uptake increased up to a concentration of 1 mM; however, the expression decreased at the highest concentration of 10 mM H_2_O_2_ (Figure 7C). PI seemed to have marginally greater statistical significance with respect to the differences obtained between groups. Similar patterns of expression were also obtained for the PI and ANV-FITC staining of hCMEC/D3 spheroids after the induction of cell death with ionizing radiation (Supplementary Figure S5). ANV-FITC showed a linear relationship to the radiation dose, while PI peaked at intermediate doses. Overall, the combination of viability and cell death dyes demonstrated that these endothelial spheroids are predominantly viable, with a higher proportion of either necrotic or apoptotic cells at the core.

## 4. Discussion

The goal of this study was to establish an endothelial model of 3D spheroids and establish simple mechanisms to achieve and assess mosaic gene expression, with a view to future use in the study of myriad mosaic driver mutations relevant to brain AVMs. Other groups have used 3D endothelial spheroids to investigate cerebral cavernous malformations (CCMs), but the use of CRISPR/Cas9 to reproduce germline mutations [29,30] is not reflective of the post-zygotic nature of the mutations common to sporadic brain AVMs.

We assessed two different endothelial cell lines for their ability to form 3D structures. Using a simple aggregation approach on non-adherent plates, we found that hCMEC/D3 cells could spontaneously aggregate to form robust reproducible spheroids, while HUVEC-TERT2 required addition of soluble type I collagen to the culture medium. It is unclear why HUVEC-TERT2 could not spontaneously aggregate, but multiple studies highlight the need for matrix components such as collagen, or thickening agents such as methylcellulose, for endothelial or tumor spheroid formation [2,18]. The hCMEC/D3 cell line has traditionally been utilized to study the blood–brain barrier as it retains tight barrier functions and transporter expression even in the absence of co-cultured VSMC or astrocytes [31,32]. For this reason, it is often used in studies examining transcytosis and drug delivery to the brain [7,33,34]. Tight junction formation and robust cell–cell adhesion may contribute to this spontaneous aggregation. Endogenous basement membrane production could also be a factor that overcomes the need for exogenous matrix supplementation, and indeed, using immunostaining, we demonstrated that the hCMEC/D3 spheroids were able to spontaneously produce collagen I. In the HUVEC-TERT2 spheroids, the supply of exogenous collagen appears to contribute to the formation of a semi-rigid basement membrane around the spheroid; however, it could not provide extended spheroid stability, based on observations that the spheroids formed from this cell line tended to “burst” rather than grow uniformly in size when cultured for an extended time or with the addition of growth-stimulating supplements. Presumably, the rupture represents “escape” of proliferating cells at the periphery, the cells unable to autogenously regulate cell–cell adherence under these conditions.

The hCMEC/D3 spheroids could be formed in a highly reproducible manner, expressed the endothelial marker CD31 at the surface, and demonstrated slow detectable growth for up to 4 weeks without spheroid rupture, consistent with the early published studies of Korff and Augustin using primary ECs [2]. Growth could be easily monitored and quantitated in the Incucyte SX5 live-cell imaging system, which provided a clear view of spheroid morphology at all stages. These spheroids demonstrated the presence of necrotic or apoptotic cells in the spheroid core within a few days of establishment. We used PI and 7-AAD as permeating dyes to assess the apoptosis and necrosis in the core. Both dyes are able to enter damaged cells and bind DNA. PI, in particular, has shown good permeation into tumor spheroids to provide reliable data on cell viability in a 3D context and in response to drug treatments [35,36,37]. We further validated these dyes by inducing ROS formation with hydrogen peroxide or radiation, demonstrating increasing uptake of both dyes in damaged cells, reflective of the morphological changes and spheroid disruption observed visually. Interestingly, the dye uptake was biphasic. Similar studies in tumor spheroids subject to radiation treatment demonstrated this same biphasic response with PI [36]. The latter study utilized live-cell imaging to assess the dye profiles, which was far more informative, and further use of these dyes to assess cellular damage should only be considered in live-cell imaging studies that produce time-dependent curves rather than static measurements as used in this study. ANV-FITC appeared to be a better marker of the induction of cell death and remained linear within the ranges tested for both radiation and H_2_O_2_. ANV-FITC can bind early apoptotic cells that expose phosphatidyl serine at the cell surface but also late apoptotic cells that have become significantly permeabilized and allow access of the ligand to PS on the cytoplasmic side of the membrane [26]. Combinations of PI/7-AAD and ANV-FITC are most often used to differentiate cells in early and late apoptosis from necrotic cells in flow cytometry [27,28]. Staining the spheroids with Annexin V-FITC showed that there was a lack of apoptotic cells on the surface in the untreated spheroids, consistent with the PI uptake data, but that binding increased the linearly with the hydrogen peroxide (or radiation) dose. It is unclear, however, whether this molecule could penetrate the spheroid to label internal cells but could make a suitable marker for cell surface analysis in a 3D context. Calcein-AM has also been utilized as a dye with good permeation of tumor spheroids for the assessment of viability in a 3D context [35], and here, it showed an appropriate inverse relationship with the PI and 7-AAD uptake in the untreated and damaged spheroids. Use of the commercial CyQUANT kit further validated the distribution of viable cells. Korff and Augustin also found that with the extended culture of endothelial spheroids, the core became acellular [2], and we also observed this in the hCMEC/D3 spheroids after extended growth using DAPI staining. The inability to completely trypsinize and digest established hCMEC/D3 spheroids also supports the hypothesis that the acellular core is predominantly extracellular matrix, lacking digestible cell–cell adhesions. Overall, the spheroids formed with hCMEC/D3 cells by this simple methodology appeared to represent a valid endothelial model to take forward.

As noted, previous studies have used 3D endothelial spheroids to investigate germline mutations in CCM, but their methodology with 100% mutation penetration may not be the most appropriate for disorders with mosaic mutations, as found in brain AVMs. Recent in vivo studies have suggested that brain AVMs driven by KRAS mutations are affected by the depth of the mutant penetration in the endothelium (that is, larger AVMs have a proportionally larger KRAS-mutant population) [11]. Thus, a model to investigate K-RAS mosaicism could potentially provide a greater understanding of the contribution of this characteristic to brain AVM development, and transient AAV gene delivery was considered an appropriate approach. To first establish this mosaic expression pattern, the AAV uptake in the EC lines was investigated with serotypes previously established to infect ECs [19,20,21]. AAV transduction did not affect the cell viability in 2D cultures and did not interfere with the spheroid formation. AAV1 and AAV2^QUADYF^ demonstrated excellent transduction efficiency in both cell lines; however, the AAV5 uptake was very poor. AAV2^QUADYF^ has a genetically modified capsid for specific endothelial uptake and may prove useful in any future mixed culture adaptations incorporating VSMC [21]. Both approaches to transduction (addition pre- and post-spheroid formation) produced mosaic expression. Transduction after spheroid formation resulted in a more random distribution of eGFP expression, which appeared clustered and may be more likely to represent the random patterns of mosaic gene expression deriving from somatic cell mutation. Extended culture of AAV2^QUADYF^-transduced, eGFP-expressing spheroids showed that hCMEC/D3 spheroids could maintain stable expression for at least 4 weeks, providing a stable model for future assessment of mosaic gene expression. The ability of 3D spheroids to facilitate prolonged survival and cell and gene maintenance during extended culture, in the absence of constant media refreshment (as required in 2D cultures), means future studies using low-level mosaic gene expression may be able to detect subtle phenotypic changes in response to mutations or drug treatments not detectable in 2D cultures. Automated live-cell imaging with the Incucyte SX5 would also increase our ability to detect subtle changes in response to mutations or drug treatments in a more sensitive, high-throughput approach.

## 5. Conclusions

In summary, we have used hCMEC/D3 cells and simple aggregation in non-adherent multi-well plates with AAV transduction to establish a robust 3D spheroid model to study endothelial mosaic gene expression. We have established dyes most suitable for manual or rapid high-throughput assessment of spheroid viability and responses to cell-damaging agents. This simple approach should be suitable for any study investigating endothelial responses to mosaic mutations, drug interventions or radiation.

## Data Availability

Raw data are available on reasonable request.

## Supplementary Materials

The following supporting information can be downloaded at https://www.mdpi.com/article/10.3390/jcdd11100305/s1, Video S1: Live-cell imaging of hCMEC/D3 spheroid formation; Videos S2 & S3: Live-cell imaging of HUVEC-TERT2 spheroid formation with and without collagen; Figure S1. Analysis of spheroid formation in home/D3 cells; Figure S2. Technical replicates demonstrating reproducibility of hCMEC/D3 spheroid formation between independent experiments; Figure S3. Z-stack series showing eGFP expression throughout an hCMEC/D3 endothelial spheroid; Figure S4. 3D rendering of Z-stack images from hCMEC/D3 endothelial spheroid after 3 weeks of growth; Figure S5. Effect of radiation on cell death in hCMEC/D3 spheroids.

## Abbreviations

7-AAD: 7-aminoactinomycin D; AAV, adeno-associated virus; ANV, annexin V; AVM, arteriovenous malformation; CCM, cerebral cavernous malformation; CMV, cytomegalovirus; EC, endothelial cells; eGFP, enhanced green fluorescent protein; FBS, fetal bovine serum; FITC, fluorescein isothiocyanate; hCMEC/D3, human cerebral microvascular endothelial cells (D3); HUVEC, human umbilical vein endothelial cells; K-RAS, Kirsten rat sarcoma viral oncogene homologue; PI, propidium iodide; ROS, reactive oxygen species; TERT2, telomerase reverse transcriptase; VSMC, vascular smooth muscle cells.
