# Supplementary material for: A Simple Model to Study Mosaic Gene Expression in 3D Endothelial Spheroids
Source: J Cardiovasc Dev Dis. 2024 Oct 2;11(10):305. doi: 10.3390/jcdd11100305 (PMC11508842; doi:10.3390/jcdd11100305)
Supplement: Supplementary file 1 [file jcdd-11-00305-s001.zip › jcdd-3072126_supplementary materials_Figures.pdf]

SUPPLEMENTARY MATERIALS

A simple model to study mosaic gene expression in 3D endothelial spheroids

Lucinda S. McRobb[1]*, Vivienne S. Lee[1], Fahimeh Faqihi[1] and Marcus A. Stoodley[1]

1 Macquarie Medical School, Faculty of Medicine, Health, and Human Sciences, Macquarie University, Sydney, New South Wales, Australia

* Correspondence: Lucinda.mcrobb@mq.edu.au; +61 2 9850 2707

Video S1: hCMEC/D3 spheroid formation

Video S1: Live-cell imaging of hCMEC/D3 spheroid formation. hCMEC/D3 cells were seeded at 1250 cells per well into Nuncla Sphera non-adherent U-bottom 96-well plates (Thermofisher). Cells were monitored using an Incucyte SX5 (Sartorius) every 6 h for 10 days and a video created with consecutive brightfield images (2s per frame). The video shows cells pooling quickly at the bottom of the U-shaped well before aggregating, with spheroid formation reproducibly occurring between days 2 to 3. The spheroids formed by hCMEC/D3 cells can move freely in the well and are robust, able to be transferred between plates or vehicles.

Video S2: HUVEC-TERT2 do not forms spheroids in the absence of collagen

Video S3: HUVEC-TERT2 spheroid formation in the presence of collagen

Video S2 & S3: Live-cell imaging of HUVEC-TERT2 spheroid formation with and without collagen. HUVEC-TERT2 endothelial cells were seeded at 10,000 cells per well into Nuncla Sphera non-adherent U-bottom 96-well plates (Thermofisher). Cells were monitored using an Incucyte SX5 (Sartorius) every 6 h for 7 days and videos created with consecutive brightfield images (2s per frame). Video S2 shows that in the absence of collagen I in the growth medium the HUVEC-TERT2 endothelial cells do not aggregate to form spheroids. Video S3 shows HUVEC-TERT2 cells pooling quickly at the bottom of the U-shaped well before aggregating, with spheroid formation reproducibly occurring within 1-2 days after seeding in the presence of 10 µg/ml rat tail collagen I.

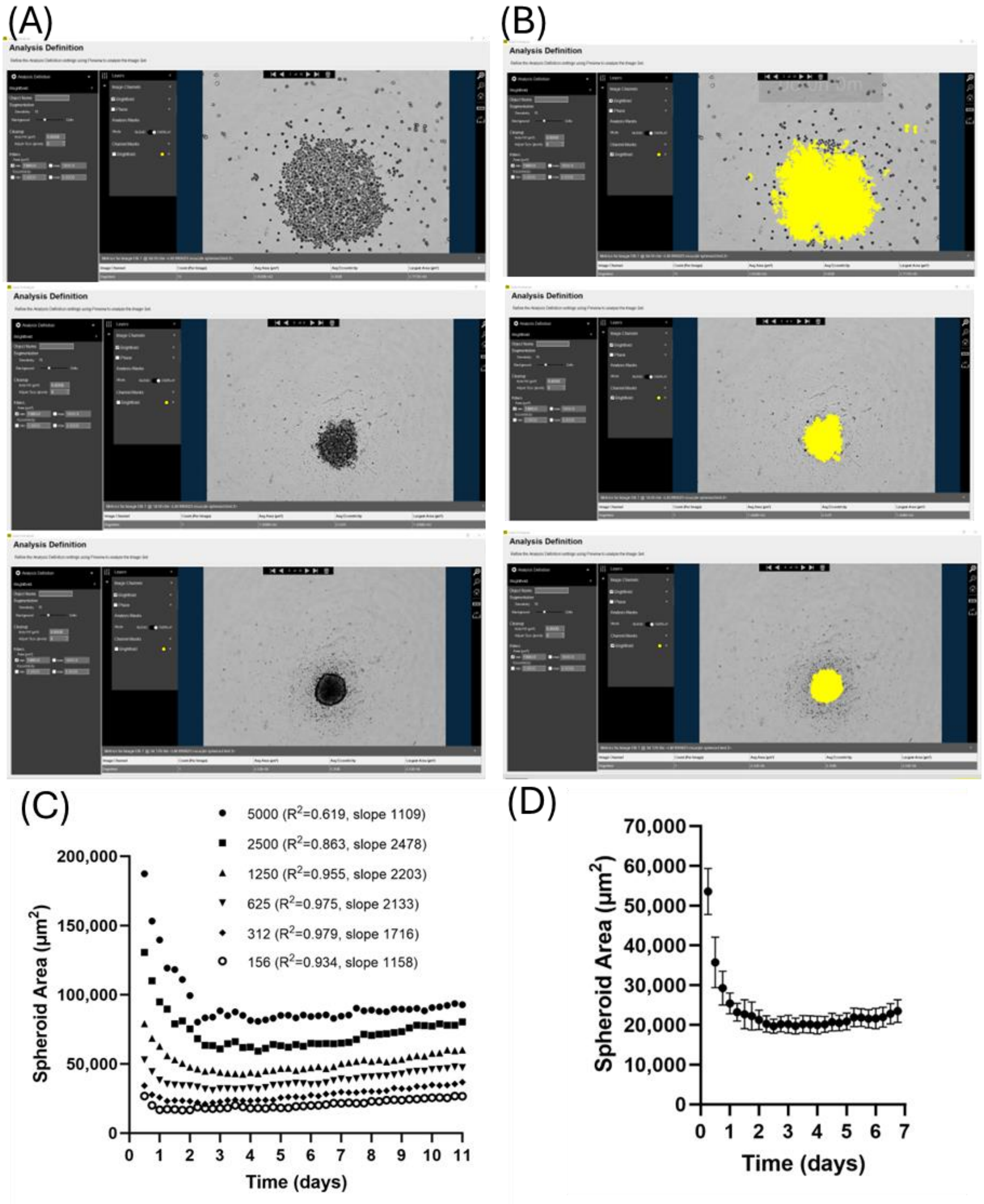

Figure S1. Analysis of spheroid formation in hCMEC/D3 cells. a) Representative screen shots from the Incucyte SX5 software showing a series of images in U-bottom wells as spheroids form. Upper, middle and lower images show the cells at 0, 1 and 3 days after seeding in non-adherent plates. At day 0, the cells have only pooled at the bottom of the wells, but by day 1 are starting to aggregate spontaneously. b) Mask creation was performed in the Incucyte SX5 software. Images demonstrate how thresholding was used to create masks (in yellow) to define the spheroid area. Note that if this was applied prior to spheroid formation (upper image) the area measured represented the total area occupied by the settling cells rather than spheroid size. This is shown in the graphs at c) and d) where spheroid area appeared to be decreasing over the first 1-2 days when measured by the Incucyte SX5. This artifact meant careful analysis of data was required to ensure measurements were valid. The ability to view live images enabled this validation. c) This graph is an extension of Fig.1c showing all data from the start of the experiment with hCMEC/D3 seeded at densities between 156 and 5000 cells per well and immediately placed in an Incucyte SX5 Imaging System for live cell imaging analysis. Mean area of largest brightfield images (μm^2) was determined for 6 technical replicates per cell seeding density (SD not shown for clarity). Simple linear regression was performed on data from day 3 to 11

(after stable spheroids formed) to determine best fit (slope = growth rate at μm²/day) and goodness of fit (R^2) for each seeding density. (d) Example of single experiment data from Incucyte SX5 with replicate spheroids (seeded at 500 cells per well) first pooling at the bottom of the well before aggregating into a tight sphere (days 1-2) before spheroid formation around day 3. (N=16 technical replicates, single plate reproducibility, mean ± SD).

(A) 500 cells seeded per well seeded at day 1 (N=4 technical replicates)

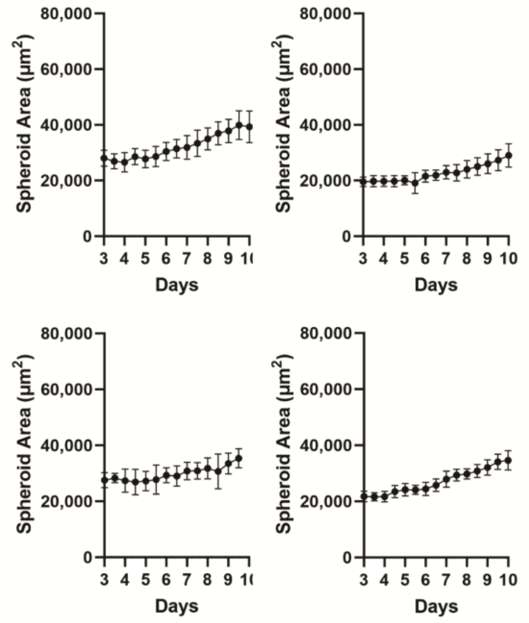

(B) 1000 cells seeded per well seeded at day 1 (N=4 technical replicates)

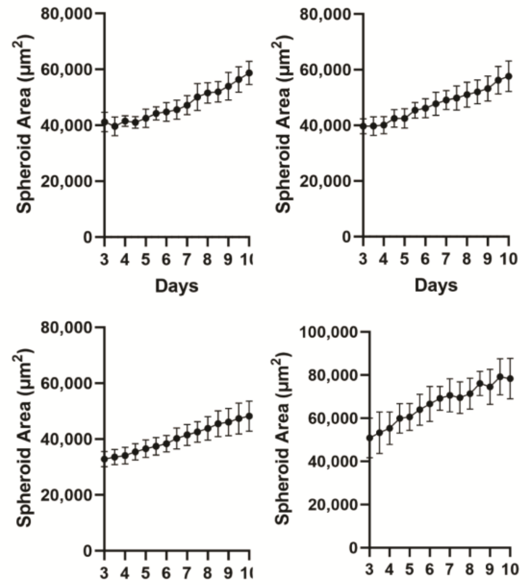

Figure S2. Technical replicates demonstrating reproducibility of hCMEC/D3 spheroid formation between independent experiments. Graphs of data for 4 independent experiments at each seeding density. hCMEC/D3 cells were seeded at day 1 with either 500 cells per well (A) or 1000 cells per well (B) with pooling and aggregation occurring between days 2-3. By day 3 cells reproducibly aggregated into spheres with clearly defined borders. Spheroids were then monitored by live-cell imaging with the Incucyte SX5 every 12 h. Spheroid area (μm²) was determined with Incucyte Software after "mask" creation for n=14 spheroids per plate (series A) or N=6-8 spheroids per plate (series B).

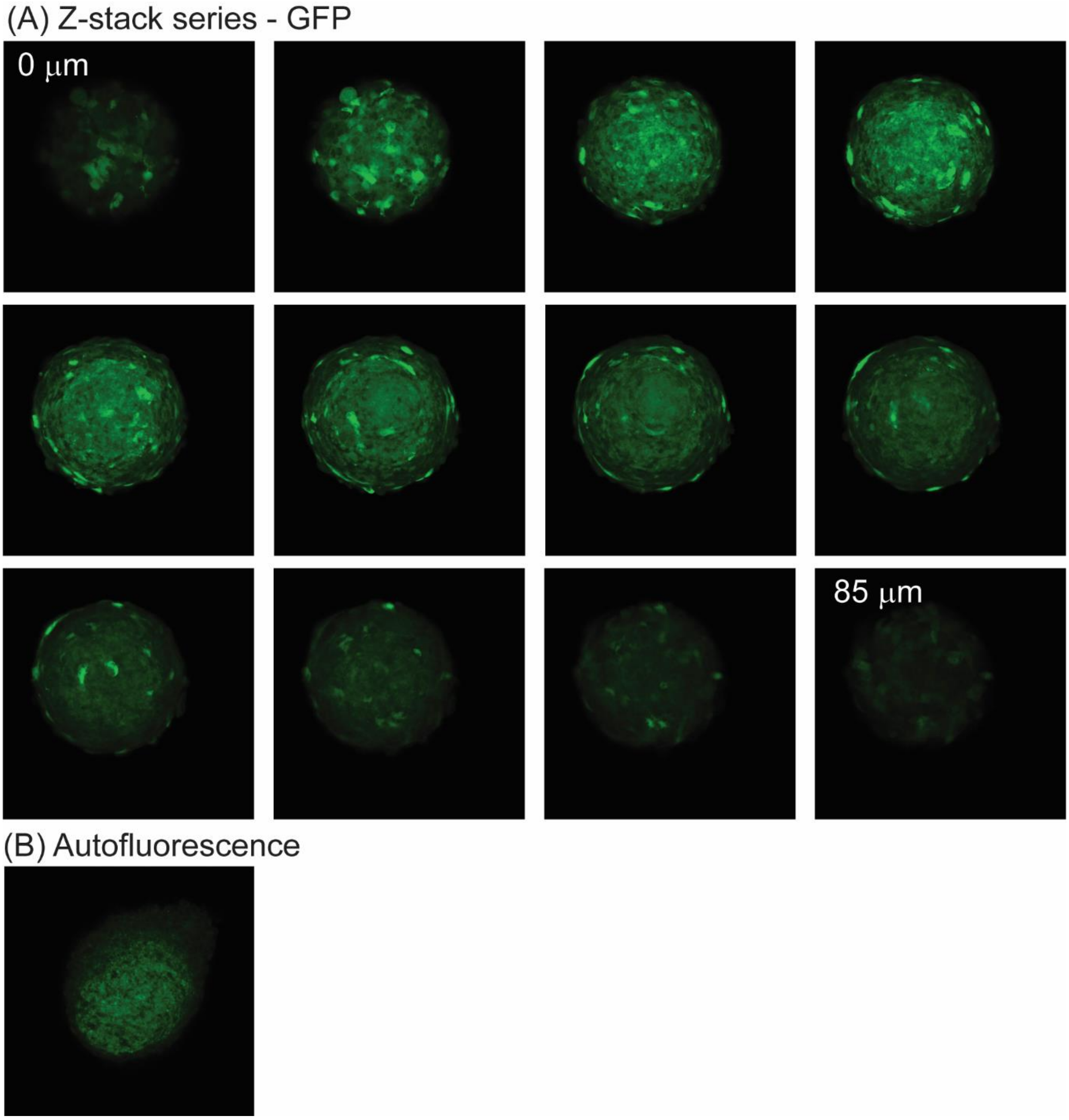

Figure S3. Z-stack series showing eGFP expression throughout an hCMEC/D3 endothelial spheroid. (a) hCMEC/D3 cells were seeded at 1000 cells per well and after 3 days, once spheroids had formed, AAV2^{QuadYF}-eGFP particles were added. Spheroids were left for 23 days before fixation and immunostaining. Confocal microscopy (Zeiss Confocal LSM880, 20x objective) was performed by fixing the first (0 μm) and last sections (85 μm) and capturing images at equal intervals. eGFP (green) expression was distributed in a mosaic pattern throughout the spheroids, both centrally and at the periphery. (b) Autofluorescence (green) was demonstrated under the same settings in the central region of all spheroids in the absence of AAV transduction.

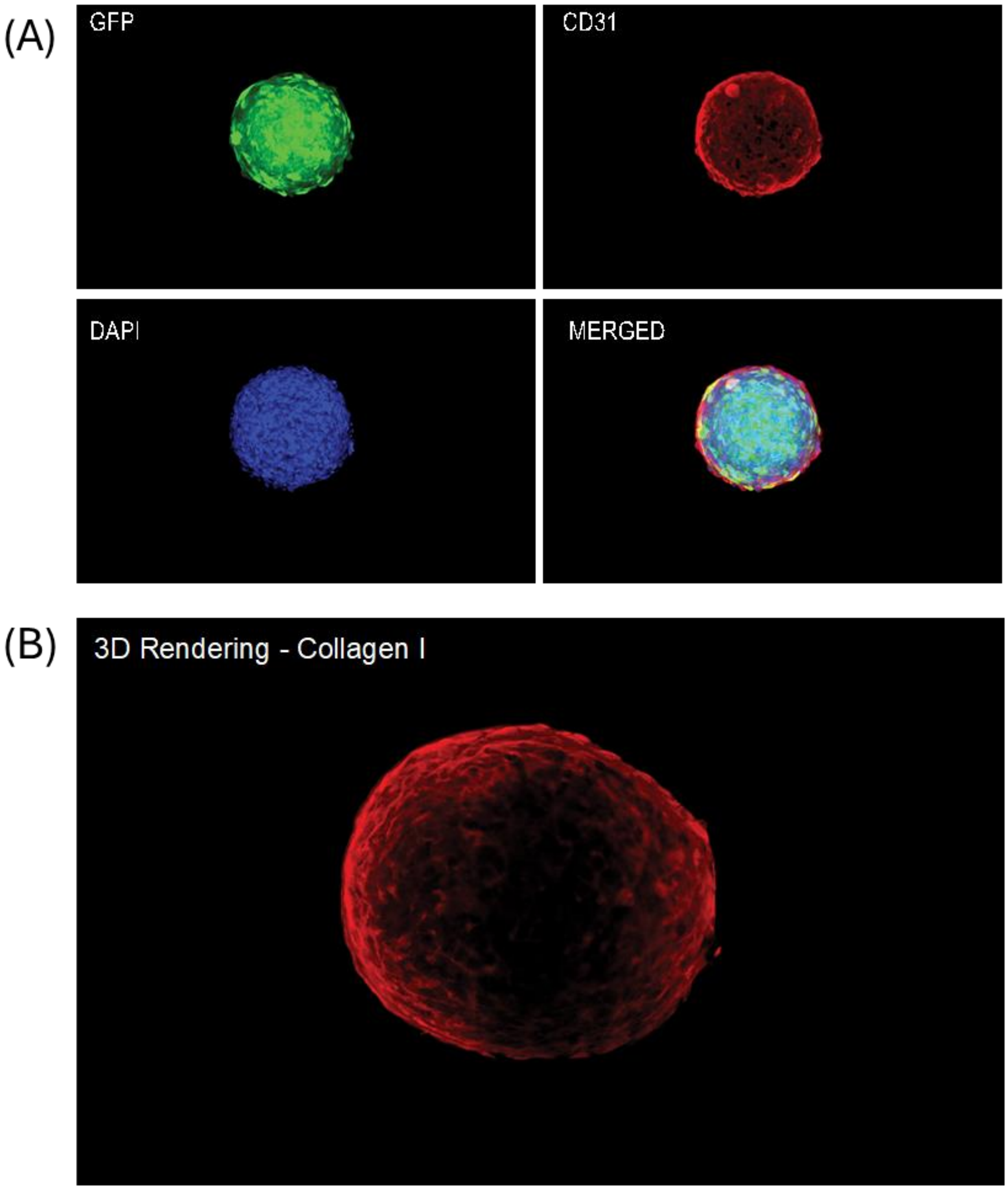

Figure S4. 3D rendering of Z-stack images from hCMEC/D3 endothelial spheroid after 3 weeks of growth. (a) 3D rendering of Z-stack images immunostained with an anti-D31 antibody CD31 (AF647, red) and transduced with AAV2-eGFP (green) and stained with the nuclear dye, DAPI (blue). (b) 3D rendering of Z-stack images immunostained using an antibody to Collagen I (AF647, red). Images were collected on a Zeiss Confocal LSM880 Microscope, 20x objective.

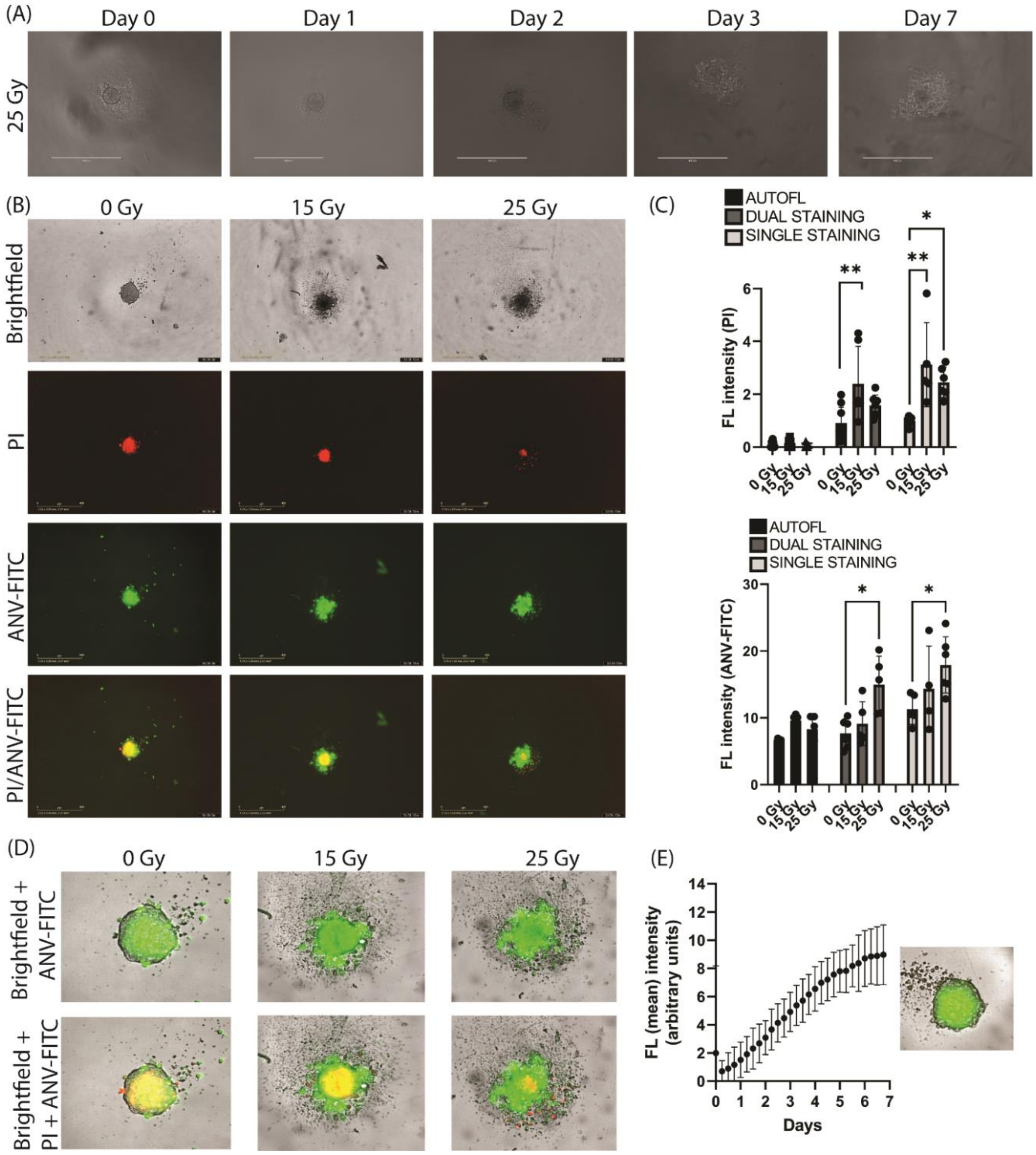

Figure S5. Effect of radiation on cell death in hCMEC/D3 spheroids. hCMEC/D3 spheroids at day 3 post-seeding were subject to irradiation using a 6MV linear accelerator and single doses of 15 or 25 Gy. PI and ANV-FITC were added to irradiated cells 1 day after irradiation to monitor cell death. Spheroids were monitored by EVOS FL Microscope or Incucyte SX5 Live Cell Imaging System 2 h after dye addition. Dyes were added either individually or in combination. (a) Representative images of hCMEC/D3 spheroids from the EVOS FL Inverted Microscope subject to ionizing radiation (25 Gy). Spheroids disintegrated over a period of 7 days to form a solid core and halo of cells at the periphery. (b) Representative brightfield and fluorescent live-cell images (ANV-FITC, green; PI, red; yellow co-localization) of hCMEC/D3 spheroids 1 day after irradiation. PI uptake was greatest at the centre of the spheroid, while ANV-FITC bound predominantly at the surface in irradiated cells. PI staining was maximal at 15 Gy, while ANV-FITC staining showed a linear response to dose. (c) Fluorescence intensity (mean intensity) was determined in staining sets which included analysis of each of the dyes when added to spheroids independently (PI or FITC, single staining) or when added in combination (PI/FITC, double staining). All data represent mean ± SEM of 3 independent experiments. One-way

ANOVA was used for statistical comparisons with Tukey's post-hoc test; *$P<0.05$, **$P<0.01$. (d) Enlarged images from Incucyte SX5 showing ANV-FITC (green) and PI staining (red) on brightfield background. Yellow represents green/red co-localization. (e, inset) Autofluorescence was significant in the green channel but not the red channel as shown by the representative Incucyte SX5 image showing high green autofluorescence emanating from the center of the spheroid in the unstained control, but no red autofluorescence (100 ms exposure). (e, graph) Green autofluorescence increased steadily with time in these spheroids (Representative experiment, n=8 spheroids, mean ± SD).
